# Somatostatin receptor scintigraphy in patients with rheumatoid arthritis and secondary Sjögren’s syndrome treated with Infliximab: a pilot study

**DOI:** 10.1186/s13550-016-0202-y

**Published:** 2016-06-04

**Authors:** L. K. Anzola-Fuentes, M. Chianelli, F. Galli, A. W. J. M. Glaudemans, L. Martin Martin, V. Todino, A. Migliore, A. Signore

**Affiliations:** Nuclear Medicine Unit, Clinica Reina Sofia, Bogotà, Colombia; Department of Nuclear Medicine and Molecular Imaging, University Medical Centre Groningen, University of Groningen, Groningen, The Netherlands; Nuclear Medicine Unit, Department of Diagnostic Imaging, Regina Apostolorum Hospital, Albano, Rome, Italy; Nuclear Medicine Unit, Faculty of Medicine and Psychology, “Sapienza” University, Rome, Italy; Rheumatology Unit, Department of Internal Medicine, Regina Apostolorum Hospital, Albano, Rome, Italy; Division of Internal Medicine, Ospedale Fatebene Fratelli S. Pietro, Rome, Italy; Nuclear Medicine Unit, Department of Medical-Surgical Sciences and of Translational Medicine, Sapienza University of Rome, Rome, Italy

**Keywords:** Rheumatoid arthritis, Sjögren, Inflammation imaging, ^99m^Tc-EDDA/HYNIC-TOC, Somatostatin receptor scintigraphy, Infliximab

## Abstract

**Background:**

Human T lymphocytes infiltrating tissues in autoimmune diseases are known to express somatostatin receptors amongst other activation markers. In this study, we evaluated whether somatostatin receptor scintigraphy (SRS) using a radiolabelled somatostatin analogue (^99m^Tc-EDDA/tricine-HYNIC-tyr(3)-octreotide (^99m^Tc-EDDA/HYNIC-TOC)) is able to detect the presence of immune-mediated processes in patients with rheumatoid arthritis and secondary Sjögren’s syndrome. We also aimed to evaluate whether positivity to SRS was predictive of therapeutic response and if SRS could be used for monitoring the efficacy of immunomodulatory treatment.

**Methods:**

Eighteen patients with rheumatoid arthritis and secondary Sjögren’s syndrome not responding to conventional treatment were recruited for treatment with infliximab, a monoclonal antibody against TNF-α. All patients had complete blood cell count, renal and liver function tests, measurements of ESR, CRP, ANA, ENA, and anti-dsDNA antibodies, functional salivary gland scintigraphy, labial biopsy, and ophthalmologic assessment with Schirmer’s test and tear film break-up time (BUT). Diagnosis was made according to the revised criteria of the American-European Consensus Group. All patients underwent SRS at baseline and after 3–6 months of therapy with infliximab. Eleven out of 18 had repeat SRS images. Images of the salivary glands and major joints were acquired 3 h after injection of 370 MBq of ^99m^Tc-EDDA/HYNIC-TOC. Image analysis was performed semi-quantitatively.

**Results:**

All patients showed uptake of ^99m^Tc-EDDA/HYNIC-TOC in the joints. Salivary glands also showed variable radiopharmaceutical uptake in 12 out of 18 patients, but all patients showed presence of lymphocytic infiltration at labial salivary gland biopsy. All patients, who repeated the study after treatment, showed significant reduction of somatostatin uptake in the joints but not in the salivary glands.

**Conclusions:**

SRS using ^99m^Tc-EDDA/HYNIC-TOC may be a useful imaging tool to assess disease activity and extent in patients with rheumatoid arthritis and may help to detect secondary Sjögren’s syndrome. It may also aid therapy decision-making with anti-TNFα antibodies in the joints but not in salivary glands.

## Background

Sjögren’s syndrome (SS) and rheumatoid arthritis (RA) are chronic inflammatory autoimmune diseases that may frequently coexist in affected patients. The former is characterised by a decrease in lacrimal and salivary secretion. It can be primary (idiopathic) or secondary (when associated with RA, ankylosing spondylitis, systemic lupus erythematosus, and others). SS is particularly relevant amongst autoimmune diseases because of its high incidence and unknown aetiology. In particular, secondary SS may be present in up to 30 % of patients with systemic lupus erythematosus and up to 20 % of patients with RA. [1]. The majority of affected patients are females (90 %), aged between 40 and 60 years. This might be related to the immunoregulatory properties of the sex hormones; however, some genetic and environmental factors may also play a role [2]. The causes of SS have not been elucidated yet, but it is always characterised by a lymphocytic infiltration in the exocrine glands (mainly the salivary and lacrimal glands) and the presence of circulating autoantibodies that advocate for autoimmune phenomena. Cytokines derived from both T and B lymphocytes contribute to the destruction of glandular tissue and inflammation [3].

An important mediator of chronic inflammation in both RA and SS is the tumour necrosis factor alpha (TNF-α) which is a cytokine with stimulating or inhibiting activity directly on immune cells. Impairment of TNF-α production causes pro-inflammatory effects through the production of many cytokines, such as interleukin-8 [4]. Given its role in autoimmune disorders, therapeutic approaches based on its blockage has been proposed. In particular, anti-TNF-α monoclonal antibodies (mAbs) such as infliximab or adalimumab have been used in patients affected by RA with positive results. Nowadays, infliximab is approved for the treatment of moderate to severe active RA, Crohn’s disease, ulcerative colitis, ankylosing spondylitis, psoriatic arthritis, and plaque psoriasis and is also prescribed (off label) for the treatment of Behçet’s disease and sarcoidosis [5]. However, its use in SS showed controversial results and recent trials in patients have failed to confirm any benefit of this therapy [6]. Similarly, it has been reported that not all RA patients respond to therapy with anti-TNF-α antibodies [7]. This led to the hypothesis that not all inflammatory processes in patients affected by RA and SS are mediated by TNF-α. Therefore, non-invasive tools to evaluate its presence in inflamed lesions would help clinicians in selecting patients who could benefit from infliximab therapy [8].

Over the last few years, somatostatin receptor scintigraphy (SRS) using somatostatin analogues has been widely used in diagnosing different types of inflammatory diseases, such as Graves’ ophthalmopathy, granulomatous diseases, and rejection of cardiac allografts and in the formation of vulnerable atherosclerotic plaques [9]. Indeed, somatostatin has regulatory effects on immune cells, associated with T cell function, and inhibits the production of cytokines such as TNF-α, IL-1, and IL-6 [10]. Therefore, since somatostatin receptor type 2 is overexpressed by activated lymphocytes in chronic immune-mediated diseases [11], it could be a potential target for peptide receptor imaging.

The aim of this study was to evaluate whether SRS is capable of detecting the presence of immune-mediated processes in patients with RA and secondary SS before and after immunomodulatory therapy with infliximab. In addition, we investigated the effect of treatment on the function of salivary glands.

## Methods

### Patients

Scintigraphy with ^99m^Tc-EDDA/tricine-HYNIC-tyr(3)-octreotide (^99m^Tc-EDDA/HYNIC-TOC) was performed in 18 patients (2 males and 16 females; age range 18–70 years; mean age 46.5 ± 12.2 years) affected by both RA and secondary SS, resistant to conventional treatment and diagnosed according to the revised criteria of the American-European Consensus Group for SS [12]. In all patients, complete blood cell count, renal and liver function tests, erythrocyte sedimentation rate (ESR), C-reactive protein (CRP), antinuclear antibodies (ANA), extractable nuclear antigens (ENA), anti-dsDNA antibodies, labial biopsy, and ophthalmologic assessment with Schirmer’s test and tear film break-up time (BUT) were performed. At the time of the study, all patients were receiving immunomodulatory drugs (cortisone and/or cyclosporine A and/or metrothexate).

Three to 6 months after the end of the treatment with infliximab (protocol used in the ATTRACT study) [13], the SRS was repeated in 11 patients to evaluate the effect of the therapy on the inflammatory process in affected joints and salivary glands. Salivary gland scintigraphy (SGS) was also performed to evaluate the functional status of salivary glands pre- and post-treatment.

In addition, 20 patients with neuroendocrine tumours (NETs), but without inflammatory lesions in joints and salivary glands, were included for ^99m^Tc-EDDA/HYNIC-TOC scan to investigate the uptake in those sites.

The study was approved by the Clinica Reina Sofia, Bogotà and Regina Apostolorum Hospital, Rome and has been performed in accordance with the ethical standards as laid down in the 1964 Declaration of Helsinki and its later amendments or comparable ethical standards.

### Radiopharmaceutical

^99m^Tc-EDDA/HYNIC-TOC, a somatostatin analogue labelled with ^99m^Tc was used for SRS. This radiopharmaceutical binds with high affinity to type 2,3, and 5 somatostatin receptors [14] and was prepared from a commercially available kit (^99m^Tc-Tektrotyd, POLATOM, Otwock, Poland), according to the manufacturer’s instructions. Briefly, 740 MBq of ^99m^Tc-pertechnetate in 0.9 % NaCl solution (pH = 7) was added to a vial containing 20 μg HYNIC-Tyr^3^-octreotide, 40 μg stannous chloride(II), 50 mg tricine, 10 mg mannitol, and 10 mg ethylenediaminodiacetic acid (EDDA). The solution was gently stirred and incubated at 80 °C for 30 minutes.

### Scintigraphic imaging

Static planar images of all major joints and of the salivary glands were acquired 3 h after the i.v. injection ^99m^Tc-EDDA/HYNIC-TOC (370 MBq) for 10 min using a 512 × 512 matrix. Thirty minutes before the radiopharmaceutical injection, patients were treated with 400 mg of KClO_4_ (Pertiroid®) to prevent the uptake of free ^99m^TcO_4_^-^ possibly released by the catabolism of ^99m^Tc-EDDA/HYNIC-TOC.

Functional sialoscintigraphy (SGS) was performed on a separate day and less than 5 days after SRS, by i.v. injection of ^99m^TcO_4_^-^ (185 MBq) and acquisition of dynamic images for 32 min using a 128 × 128 matrix. Lemon juice (2 ml) was given 16 min after the radiopharmaceutical injection to evaluate salivary excretion. Time activity curves were generated to evaluate the uptake and secretion pattern. Images were acquired with a double-headed gamma camera (Philips Forte, The Netherlands) equipped with a low-energy high-resolution collimator.

### Image analysis

Evaluation of each ^99m^Tc-EDDA/HYNIC-TOC scan was performed visually and semi-quantitatively by two experienced nuclear medicine physicians (KAF and MC), who were unaware of the underlying pathology, patient’s clinical history, and the results of the other clinical parameters. Corresponding studies were compared for the final analysis and ruled as matching or mismatching.

The SRS was semi-quantitatively analysed in joints using a scale of 0 to 5 using the uptake in the calf muscle as the background signal where 0 corresponds to no uptake, less than background (T/B < 0.8); positivity was defined by a score from 1 to 5 with a joint having a score of 1 if uptake was detectable but lower than background (T/B between 0.8 and 1.0); 2 if uptake was similar to background (T/B between 1.0 and 1.2); 3 if uptake was slightly higher than background (T/B between 1.2 and 1.4); 4 if uptake much higher than background (T/B between 1.4 and 1.6); and 5 if uptake was clearly high (T/B > 1.6). This scale provides sufficient stratification to define the severity of the inflammatory process and enables comparison between studies and organs. The scores reported in tables are the average between scores of two readers (KAF and MC), and in particular:

- *n* = number of positive joints (with a score greater than 1),
- *global score* = the sum of all joint scores,
- *severity index* = global score divided by number of positive joints.

The uptake in the salivary glands was also semi-quantitatively analysed as follows:

- *n* = number of positive salivary glands,
- *global score* = sum of all positive glands uptake scores (scores assigned as for joints).

In functional salivary gland scintigraphy we evaluated the following parameters:

- *Uptake score* = target to background ratio of sum of activity at 12–16 min in both parotid glands before lemon juice administration (temporal region was taken as background) and at 28–32 min after lemon juice administration.
- *Functional score* = ratio between the uptake score before and after administration of lemon juice.

### Statistics

Differences between groups were evaluated by unpaired Student’s *t* test; intra-group variations were studied using paired Student’s *t* test. Regression analysis between different parameters was also performed.

## Results

Table 1 shows the demographic characteristics and findings of SRS in joints and salivary glands. All patients showed uptake in joints with a mean global score of 17.0 (with 8.5 affected joints per patient on average with a range of 1–20); however, only 12 patients out of 18 showed uptake in salivary glands (1 to 4 glands involved with a range of global score from 1 to 6) despite all patients having histologically proven secondary SS.

**Table 1:** Demographic characteristics and pre-therapy findings on SRS in joints and salivary glands of patients studied

	Age	Gender	Pre-therapy joints SRS		Pre-therapy salivary glands SRS	
Patient			*n*	Global score	*n*	Global score
1	18	F	13	23	4	6
2	25	F	4	18	1	2
3	40	F	5	5	2	2
4	32	F	2	2	2	2
5	45	F	1	3	1	2
6	60	F	7	13	0	0
7	22	M	10	15	0	0
8	36	F	4	8	2	4
9	29	F	4	14	0	0
10	42	F	8	14	2	6
11	40	F	10	14	0	0
12	52	F	10	20	0	0
13	54	M	10	24	1	3
14	60	F	12	22	0	0
15	62	F	8	16	2	2
16	64	F	17	29	1	2
17	39	F	20	52	2	1
18	70	F	8	14	2	4
Mean			8.5	17	1.2	1.9
±SD			4.9	11.3	1.1	1.9

Table 2 shows the semi-quantitative scores of SRS in joints in the 11 patients with pre-and post-therapy evaluation. Radiopharmaceutical uptake was significantly reduced in joints after therapy, with a statistically significant difference in the severity index, global score, and number of positive joints (*p* = 0.009, *p* = 0.001, *p* = 0.002, respectively).

**Table 2:** Pre-therapy and post-therapy on SRS in joints

	Pre-therapy scan			Post-therapy scan		
Patient	*n*	Global score	Severity index	*n*	Global score	Severity index
1	13	23	1.8	6	8	1.3
3	5	5	1.0	4	4	1.0
4	2	2	1.0	2	2	1.0
5	1	3	3.0	1	1	1.0
6	7	13	1.9	3	4	1.3
7	10	15	1.5	6	7	1.2
8	4	8	2.0	3	4	1.3
9	4	14	3.5	2	5	2.5
12	10	20	2.0	7	13	1.9
14	12	22	1.8	5	6	1.2
18	8	14	1.8	2	3	1.5
Mean	6.9	12.6	1.9	3.7*	5.2**	1.4***
±SD	4.0	7.3	0.7	2.0	3.3	0.4

The table shows values of the number of positive joints (*n*), of the global score and of the severity index in the joints of the 11 patients that performed somatostatin receptor scintigraphy (SRS) before and after treatment with Infliximab

*p = 0.002 vs pre-therapy *n*; **p = 0.001 vs pre-therapy g*lobal score*; ***p = 0.009 vs pre-therapy s*everity index*

Table 3 shows the semi-quantitative scores of SRS and SGS in salivary glands in patients pre- and post-therapy. SRS did not show any significant reduction of radiopharmaceutical uptake in salivary glands after therapy (mean global score 1.73 + 2.1 before therapy vs 1.18 + 1.25 after therapy; *p* = ns), and only three patients showed a mild improvement (patients 1, 8, and 18). SGS after therapy with infliximab was also similar to the scan before therapy (mean functional score 5.96 + 0.97 before therapy vs 6.23 + 0.91 after therapy; *p* = ns).

**Table 3:** Pre-therapy and post-therapy findings on SRS and SGS in salivary glands

	Pre-therapy scan			Post-therapy scan		
	SRS		SGS		SRS	SGS
Patient	*n*	Global score	Functional score	*n*	Global score	Functional score
1	4	6	7.5	3	3	7.5
3	2	2	5	5	2	2.5
4	2	2	4.5	2	2	4.4
5	1	1	5.4	1	1	6.3
6	0	0	6.2	0	0	6.3
7	0	0	5.2	0	0	6.1
8	2	4	5.7	2	2	6.5
9	0	0	6.5	0	0	6.7
12	0	0	7.6	0	0	7.4
14	0	0	6.2	0	0	6.4
18	2	4	5.8	2	3	5.9
Mean	1.18	1.73	5.96	1.09*	1.18*	6.23*
±SD	1.33	2.1	0.97	1.14	1.25	0–91

Table shows values of the number of positive joints (*n*), of the global score and of the functional score in the salivary glands of the 11 patients that performed somatostatin receptor scintigraphy (SRS) before and after treatment with infliximab

*p = n.s. vs pre-therapy values

ESR and CRP decreased significantly during the treatment period. Infliximab was well tolerated without side effect.

The review of the scans from the database of patients with NETs showed no significant peri-articular uptake around knees and shoulders with a grade score of 3 or 2 and a symmetric pattern was observed in 8 out of 20 cases, all of them older than 60 years. Uptake in hands was observed, with a score lower than 3, with a diffuse and asymmetric pattern located solely in the carpal joints. In salivary glands, there was no uptake in 16 out of 20 patients. Three patients with Hashimoto disease had positive findings in the sub-maxillary glands but not in the parotids. One patient had positive findings only in parotid with an asymmetric pattern.

## Discussion

Somatostatin receptors, amongst other markers, are known to be expressed in human T lymphocytes that infiltrate tissues in autoimmune diseases. In this study, we have used SRS using a radiolabelled somatostatin analogue (^99m^Tc-EDDA/HYNIC-TOC) to detect immune-mediated processes in patients with rheumatoid arthritis and secondary Sjögren’s syndrome. We also evaluated whether positivity to SRS was predictive of therapeutic response and if SRS could be used to monitor immunomodulatory therapy with infliximab.

**Fig. 1:**
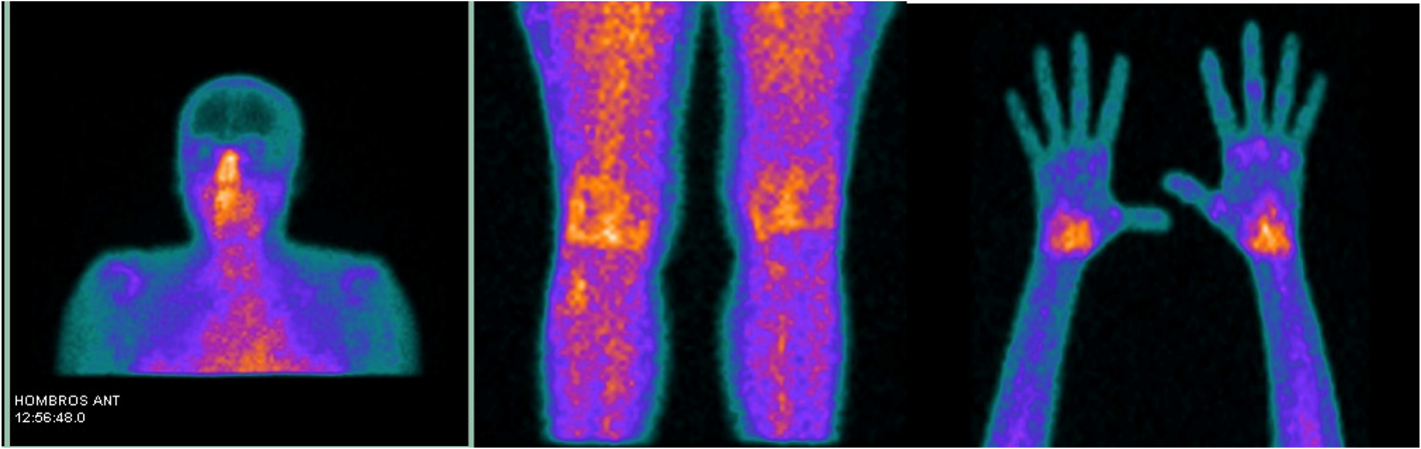
SRS of patient 17, showing high uptake of ^99m^Tc-EDDA/HYNIC-TOC in submandibular glands, shoulders, knees, and hands with symmetrical pattern in carpal and metacarpal joints

**Fig. 2:**
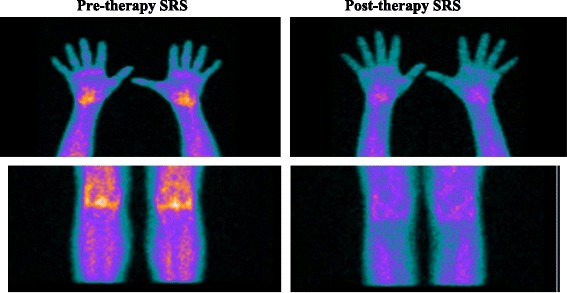
SRS in patient number 14 before (*left images*) and after therapy (*right images*) with infliximab. Global score in hands and knees before therapy was 22. Global score post-therapy was 6, showing good response to treatment

All our patients with arthritis and secondary SS ^99m^Tc-EDDA/HYNIC-TOC scans showed intense uptake with a symmetric and focal pattern in hands, predominantly in carpal, metacarpal, and proximal interphalangeal joints. This finding agrees with the 2010 ACR/EULAR criteria for diagnosis of RA in the hand [15] (Fig. 1). Other compromised joints were knees, shoulders, and ankles to a lesser degree. The quantitative analysis in all patients showed a mean global score of 17, and in the 11 treated patients the mean fell from 12.6 to 5.2 after treatment with infliximab (*p* = 0.001). Also, the mean severity index fell from 1.9 to 1.4 after therapy (*p* = 0.009) (Fig. 2). We found no correlation between joint pain or swelling and SRS positivity. Indeed, some joints that were apparently poorly affected showed high somatostatin uptake and, vice versa, in some painful and swollen joints, we found only a moderate uptake. This finding is in agreement with the theory that somatostatin receptors can be overexpressed in active phases of the disease characterised by endothelial activation and lymphocyte infiltration in the synovial cells [16]. Nevertheless, all patients and all positive joints showed a clinical and scintigraphic improvement after infliximab therapy. Thus, it can be assumed that SRS is able to identify patients with active disease responding to anti-TNF-α therapy.

A possible limitation of the present study is that patients with a negative SRS had not been treated with infliximab. Therefore, we cannot conclude that negativity at SRS is related to inefficacy of the therapeutic response. However, we can compare our results with other published studies in which patients were not selected on the basis of SRS positivity and the response to infliximab therapy had a much lower rate of success [5]. This finding supports the hypothesis that SRS could identify patients with active disease who may benefit from infliximab therapy.

In patients affected by RA, SSTRs are expressed by synovial endothelial cells and synovial macrophages. The SSTR1 and SSTR2 subtypes have been identified on rheumatoid synovial fibroblasts. The inhibitory effects of somatostatin have been documented in cultures of normal activated lymphocytes and of RA synovial cells. Somatostatin not only inhibits proliferation but also suppresses several mediators of inflammation including pro-inflammatory cytokines such as TNF-α, IL-1B, and IL-8 in vitro and in vivo [17]. Somatostatin and its receptors are produced in macrophages and lymphocytes probably via signalling of growth factors and cytokines, and co-activation has been demonstrated in T cells, granulomatous lesions, and synovial fibroblasts from RA patients [18]. In a study of 14 consecutive patients with RA, SRS demonstrated uptake of ^111^In-pentetreotide in inflamed joints with a lesion-related sensitivity of 74 % [19]. Adams et al. confirmed the expression of SSTR1 and SSTR3 on inactivated endothelial cells, whereas SSTR2 is strongly expressed after activation [20]. Diffuse infiltration of CD4^+^ lymphocytes and macrophages can always be detected in affected joints, where they express SSTR upon persistent immunological activity [21]. Studies in vitro have shown that proliferation of synovial cells of patients suffering from RA could be inhibited by somatostatin. This may provide a rationale for the therapeutic use of long-acting somatostatin analogues for treatment of this disease. In a clinical trial by Paran et al., a significant clinical improvement was observed in patients with refractory RA treated with a long-acting somatostatin analogue [22]. SRS, therefore, holds important information not only by demonstrating the presence of inflammation but, in positive patients, could also provide a rationale for the treatment of the disease with unlabelled somatostatin.

**Fig. 3:**
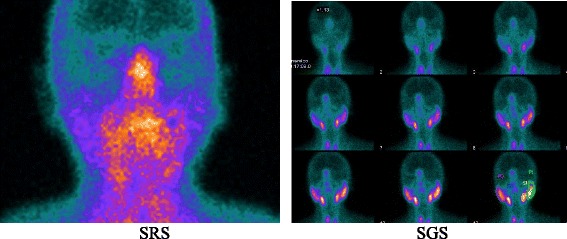
Comparison between SRS (*left image*) and SGS (*right image*) of salivary glands in the same patient. Images show the different uptake of the two radiopharmaceuticals in salivary glands. There is an evident uptake only in submandibular glands at SRS, whereas all salivary glands show uptake at SGS

Our study demonstrated that SRS scintigraphy was positive in all patients and in several different joints but not in all salivary glands. It is important to remark that when the salivary gland pattern of uptake was compared in SRS and SGS, a discrepancy was found in all positive patients. This finding confirmed that the appearance of the salivary glands in SRS is secondary to the presence of somatostatin receptors and not to free ^99m^TcO_4_^-^, which was reinforced by the fact that the stomach was not visualised in any of the patients (Fig. 3).

The analysis of the scans of patients affected by NETs and without inflammatory disease showed normal mild to moderate uptake of the radiopharmaceutical in the liver, spleen, renal shapes, and gastrointestinal tract. Faint uptake of the radiopharmaceutical was observed in the thyroid gland as reported by Duet et al. [9]. Uptake in the stomach was not observed in any patient, thus excluding the presence of circulating free ^99m^TcO_4_^-^. In some patients (>60 years old) radiopharmaceutical uptake was observed in carpal joints, knees, and shoulders with a symmetrical appearance and a score equal to or lower than 3. Vanhagen et al. postulated that this finding could be explained by osteodegenerative disorders [19]. The absence of salivary gland uptake (16 out of 20, 80 %) was a common finding. However, an increased uptake was observed in the salivary glands of three patients and was related to Hashimoto’s disease. This might be explained by the fact that the most common thyroid disorder found in association with SS is Hashimoto’s thyroiditis and that some antigens are shared by salivary glands and thyroid gland, which could be responsible for the association between these two pathologies [23].

In our study, only 12 patients of the 18 reported in this study had at least one positive salivary gland at SRS. After therapy with Infliximab (given in 11 patients, see Table 3), only 3 patients showed an improvement of salivary SRS after therapy (1, 8, and 18) and another 3 patients showed a mild improvement at SGS (patients 5, 7, and 8). There was no correlation seen between the findings of the two studies in salivary glands indicating that SRS identifies the inflamed sites mediated by somatostatin that did not necessarily correlate with the structural changes evidenced at SGS. From the clinical point of view, all patients had secondary histologically proven SS, and all patients showed clinical improvement of xerophthalmia and xerostomia after therapy with infliximab. Therefore, SRS may help to identify secondary SS in approximately 67 % of patients with RA but does not help in selecting patients who would respond to anti-TNFα. This finding agrees with a recent study in patients with primary SS in which the authors showed that anti-TNFα therapy does not have an effect on glandular and extra-glandular manifestations of SS [24]. As far as the type of treatment is concerned, to date, there is no standard treatment available for SS and no studies have been performed to evaluate the effect of any therapy on the function of infiltrated salivary glands.

Imaging techniques in SS include many different methods such as ultrasound, MRI, sialography, and salivary gland scintigraphy, but none of these techniques have a high enough sensitivity and specificity to be considered a ‘gold standard’. To date lip biopsy of the salivary glands is considered the most reliable diagnostic test and is still the most accurate for diagnosis of SS. Sensitivity and specificity of lip biopsy range from 82 to 95 % and from 75 to 90 %, respectively [25–27]. Therefore, only a combination of clinical, immunological, histological, functional, and morphological parameters can help to establish a correct diagnosis of SS and to assess the activity of the disease in order to define the most appropriate treatment and to follow-up its efficacy.

In our study, we used a ^99m^Tc-labelled somatostatin analogue (^99m^Tc-EDDA/HYNIC-TOC) to evaluate the activity status of joint inflammation and associated salivary gland inflammation in a selected group of patients with RA and secondary SS. The hypothesis behind the study was that SRS could be a useful tool for the workup of these patients for therapeutic decision-making and follow-up of biological therapies. Several hypotheses can be proposed to explain the different behaviour of joint and salivary gland disease. Firstly, the two may have a different pathogenesis and natural history. Secondly, our patients were primarily RA patients with long standing active disease and refractory to conventional therapy. The onset of SS might have occurred at different time points. Finally, it is known that secondary SS is very difficult to treat and improvement might be very poor and transient.

A possible limitation of our study could be the small number of cases that were followed up after therapy and by the absence of infliximab treatment of patients who were negative for SRS. Therefore, we cannot fully assess a correlation between positivity at SRS and therapy response. Notably, however, in other studies in which infliximab therapy had a much lower rate of success, SRS were not used to select patients for therapy [18].

## Conclusions

Our pilot study indicates that SRS using ^99m^Tc-EDDA/HYNIC-TOC is positive in joints and, to a lesser extent, in salivary glands of patients with RA and secondary SS who do not respond to conventional treatment. SRS may therefore be a useful imaging tool to assess disease activity in RA and help to detect secondary SS. Given that all positive patients showed a benefit from infliximab therapy in the joints, SRS positivity might be considered as a positive prognostic factor. We therefore suggest the use of SRS for the selection of refractory RA patients who may be treated with biological therapies but not as a tool for defining treatment in secondary SS.
